# A Structural Equation Model Analysis of Relationships among ENSO, Seasonal Descriptors and Wildfires

**DOI:** 10.1371/journal.pone.0075946

**Published:** 2013-09-24

**Authors:** Matthew G. Slocum, Steve L. Orzell

**Affiliations:** 1 Department of Biological Sciences, Louisiana State University, Baton Rouge, Louisiana, United States of America; 2 Avon Park Air Force Range, Avon Park, Florida, United States of America; DOE Pacific Northwest National Laboratory, United States of America

## Abstract

Seasonality drives ecological processes through networks of forcings, and the resultant complexity requires creative approaches for modeling to be successful. Recently ecologists and climatologists have developed sophisticated methods for fully describing seasons. However, to date the relationships among the variables produced by these methods have not been analyzed as networks, but rather with simple univariate statistics. In this manuscript we used structural equation modeling (SEM) to analyze a proposed causal network describing seasonality of rainfall for a site in south-central Florida. We also described how this network was influenced by the El Niño-Southern Oscillation (ENSO), and how the network in turn affected the site’s wildfire regime. Our models indicated that wet and dry seasons starting later in the year (or ending earlier) were shorter and had less rainfall. El Niño conditions increased dry season rainfall, and via this effect decreased the consistency of that season’s drying trend. El Niño conditions also negatively influenced how consistent the moistening trend was during the wet season, but in this case the effect was direct and did not route through rainfall. In modeling wildfires, our models showed that area burned was indirectly influenced by ENSO via its effect on dry season rainfall. Area burned was also indirectly reduced when the wet season had consistent rainfall, as such wet seasons allowed fewer wildfires in subsequent fire seasons. Overall area burned at the study site was estimated with high accuracy (*R* ^2^ score = 0.63). In summary, we found that by using SEMs, we were able to clearly describe causal patterns involving seasonal climate, ENSO and wildfire. We propose that similar approaches could be effectively applied to other sites where seasonality exerts strong and complex forcings on ecological processes.

## Introduction

In the science of ecology, seasons are generally defined as lasting fixed spans within the year. These conventional definitions, however, often ill-serve attempts to define important climate-driven relationships in ecosystems; as stated by Basille et al. [1], the use of conventional seasons can often lead to “faulty conclusions if the periods do not match biological reality.” To address this issue, over the last several decades more sophisticated methods for describing seasons have been developed, and the resulting variables related to ecological phenomena. For example, in southern Florida Slocum et al. [2] estimated how dry season rainfall affected area burned by wildfires when the season was conventionally defined versus when it was defined using cumulative rainfall anomalies. The former definition resulted in a *R* ^*2*^of 0.22 while the later resulted in a *R* ^*2*^of 0.41. In the western United States, Westerling et al. [3] used estimates of timing of snowmelt (developed by Stewart et al. [4]) to define relationships with wildfires. They found that, starting around 1987, snowmelt began to occur earlier in the year and corresponded with higher temperatures and a greater frequency of large wildfires. Finally, animal behavior scientists routinely use the behavior of animals to define seasons (e.g., [5]; see sources in [1]). Based on these examples, it is clear that ecologists are starting to view conventionally defined seasons as simplistic and information poor, and are turning to new methods to learn more about how seasons affect ecosystems.

One issue, however, that comes up when using these new methods is that the resultant data sets contain more variables than when using conventional methods. A conventional data set may, for example, contain just rainfall, whereas a more sophisticated method may estimate rainfall, onset date, cessation date, and duration. These additional variables constitute a system of seasonal components whose relationships may be complex. This complexity increases further when one considers ENSO or other global climate cycles, as such cycles may not affect just rainfall but any of the other variables describing the season [6,7]. Despite this added complexity, the relationships among the variables are usually described using univariate approaches, that is, methods that analyze just one dependent variable (e.g., correlation, multiple regression) [2,3,6,8]. This is a missed opportunity, as the relationships can be drawn as causal diagrams, for example, one that describes how seasons that begin earlier in the year are longer and have more precipitation. Once such a diagram is made, it can then be pointed towards ecological phenomena of interest. In this way, the relationships among global climate cycles, seasonality, and ecological phenomena can be described more clearly than when using univariate analyses.

Here we describe a case study in which we develop causal diagrams describing relationships among seasonal descriptors, ENSO and a wildfire regime at a subtropical site in south-central Florida (the Avon Park Air Force Range, or APAFR). The seasonal descriptors we used were derived in a previous study [2] using cumulative rainfall anomalies (CRAs). The strength of the relationships among the variables in the causal models, as well as the overall fit of the models, was estimated using structural equation models (SEMs). Our specific questions addressed by these SEMs are outlined in detail below. Using these methods, we were able to demonstrate how SEMs can be used to describe complex relationships among seasonal descriptors, as well as how these descriptors related to ENSO and an ecological disturbance regime.

## Methods

### Study Site

The APAFR is a 42,000 ha military installation in south-central Florida (27°35’ N, 81°16’ W). It has a subtropical climate that is divided into wet and dry seasons. The characterization of these seasons using CRAs [2] found that the wet season lasted, on average, from May 21 to October 1 (a duration of 134 days), while the dry season lasted from October 2 to May 20 (a duration of 231 days). The dry season, despite being twice as long as the wet season, had roughly half the rainfall (mean ± 1 SD: 42 ± 15 cm yr^-1^ versus 89 ± 27 cm yr^-1^). Year to year, there was considerable variation in how long the seasons lasted and when they started; onset dates of the two seasons had standard deviations of almost one month and durations had standard deviations greater than a month. Other studies of seasonality conducted in central and southern Florida have also found well-demarked wet and dry seasons [9-12], but none have documented annual changes in season length, onset date, or cessation date.

About 38,000 ha at the APAFR are subject to recurrent fire. The behavior of these fires is heavily influenced by subtle elevation gradients that clearly delimit plant communities of varying hydroperiod and flammability (the communities range from floodplain marshes to xeric uplands over a gradient ranging from 9.1 to 41.2 masl) [13-15]. The ground cover of these communities is dominated, to varying extent, by wire grass ( *Aristida* *beyrichiana* ), dwarf live oak ( *Quercus* *minima* ), and saw palmetto ( *Serenoa* *repens* ) [13,14]. The overstory in the pine savannas is dominated by south Florida slash pine ( *Pinus* *elliotii* var. *densa* ), longleaf pine ( *Pinus* *palustris* ), or both. While the APAFR contains tracts of managed or disturbed areas, including pine plantations, improved pastures, and target sites, it has 23,000 ha of some of the most diverse fire-maintained landscapes remaining in the region (S. Orzell, unpublished data). It also contains numerous endangered species [16].

The installation was established in World War II to provide military personnel practice in bombing, strafing, and related exercises. Because of these exercises, most of the wildfires on the range are of military origin, with a smaller portion being ignited by lightning (over 1997-2007, 89 military fires burned 15,400 ha while 51 lightning fires burned 6,350 ha; APAFR fire records). Wildfires generally occur from January to August, with the largest fires occurring during the transition between the dry and wet seasons (i.e., around May to June) (M.G. Slocum, unpublished data, cf. [11,12]). The amount of area burned by wildfires is much smaller than that burned by prescription, which accounted for 106,000 ha of the total area burned over 1997-2007. Prescribed fires are conducted to manage fuel loads and to maintain habitat for endangered species. They have been conducted on a 3 year rotation since the early 1990s. While they control fuel loads, they do not appear to substantially affect wildfires in subsequent years (Pearson correlation: area burned by prescribed fires versus area burned by wildfires in next year, *r* = 0.29, *p* = 0.30, *n* = 30 yrs). Finally, note that military and lightning wildfires tend to behave similarly in any given year (Pearson correlation: area burned by military fires versus area burned by lightning fires, *r* = 0.80, *p* = 0.003, *n* = 11 yrs).

### Data

The CRA analysis of the site’s seasons [2] used methods developed by Camberlin and Diop [8] for another subtropical region (Senegal).CRAs are useful because they convert daily rainfall data into a waveform, thereby allowing clear visualization of trends in rainfall and a full characterization of the seasons (see Appendix S1). Slocum et al. [2] specifically estimated onset date, cessation date, and duration for both the wet and dry seasons over 1950-2007 (58 years). Also, because the lengths of the seasons were rigorously defined in the analysis, rainfall was estimated more accurately than when using a conventional method for defining seasons. Lastly, the analysis provided a fifth variable, “trend consistency”, which described how consistent the drying and moistening trends were in the dry and wet seasons, respectively. This variable was measured using *R* ^2^ scores; see Appendix S1 for a brief description of how these scores were derived. Trend consistency proved to be a unique and powerful predictor of wildfire activity at the site [2]..

ENSO was described using Niño 3.4, an index derived from sea surface temperature anomalies over a specific region of the equatorial Pacific Ocean [17]. As values of this index increase, they describe increasingly stronger El Niño conditions, and as they decrease they describe increasingly stronger La Niña conditions. This index has been shown to be superior for predicting rainfall in Florida compared to other ENSO indices [18,19]. Monthly Niño 3.4 values from January 1950 to December 2007 were obtained from the National Oceanic and Atmospheric Administration’s Climate Prediction Center (information online at http://www.cpc.ncep.noaa.gov/data/indices). We took the mean of the these values over the specifically-defined spans of each season. Because these spans started and ended within months, we adjusted the calculations so that incomplete months were proportionately weighted.

Data describing number of wildfires and area burned per year were collected from the APAFR’s fire records over 30 years (1978-2007).

### Conceptual models

SEMs represent multivariate hypotheses about cause and effect in systems. They allow the evaluation of these hypotheses to discover if they are consistent with underlying patterns in the data. One issue that often comes up when discussing SEMs involves the assumption of causality among the relationships proposed. This issue is of particular concern when the data used are observational, such as in this study, because these kinds of data cannot be tested for causality using randomized experiments [20]. We therefore deem it important to state here what we mean by “causal”. When we say a relationship is causal, we mean that it is logically so (e.g., longer periods will have more rain), or are widely established to be so in the literature (e.g., ENSO affects rainfall). In cases like the latter, the causal assumption is based on a process of evidential build up: ENSO is thought to influence rainfall because the initially proposed hypothesis has been followed by numerous studies that confirmed that hypothesis [21]. Clearly, a more extensive debate of the meaning of causality is beyond the scope of this manuscript, but more information can be found elsewhere (e.g., Shipley [20]).

We developed our multivariate hypotheses by drawing on the previous modeling effort as well as on general knowledge of the system (see sources in Slocum et al. [2]). There are many ways that SEMs can be employed to better describe complex systems and to generate hypotheses to guide future studies [22]. In our study we chose to use a model building approach. We started with simple models that only contained pathways with some theoretical support. We viewed the patterns revealed by these starting models as confirming, or not confirming, theory. If these starting models did not fit the data, we then conducted an exploratory procedure in which we allowed the data to specify “competing” models within the general framework of the starting models. We then determined if these models fit the data in ways substantially different than the starting models (that is, did they simply add detail to the starting models, or did they suggest strongly differing patterns?). In the Results we detail the strength (in a statistical sense) of the various models and relationships examined, while in the Discussion we detail which patterns revealed by the SEMs are the most consistent with theory, and which appear to be more hypothesis generating. We developed separate models for wet and dry seasons, and for the APAFR’s wildfire regime.

### Seasonal models

**Figure 1:**
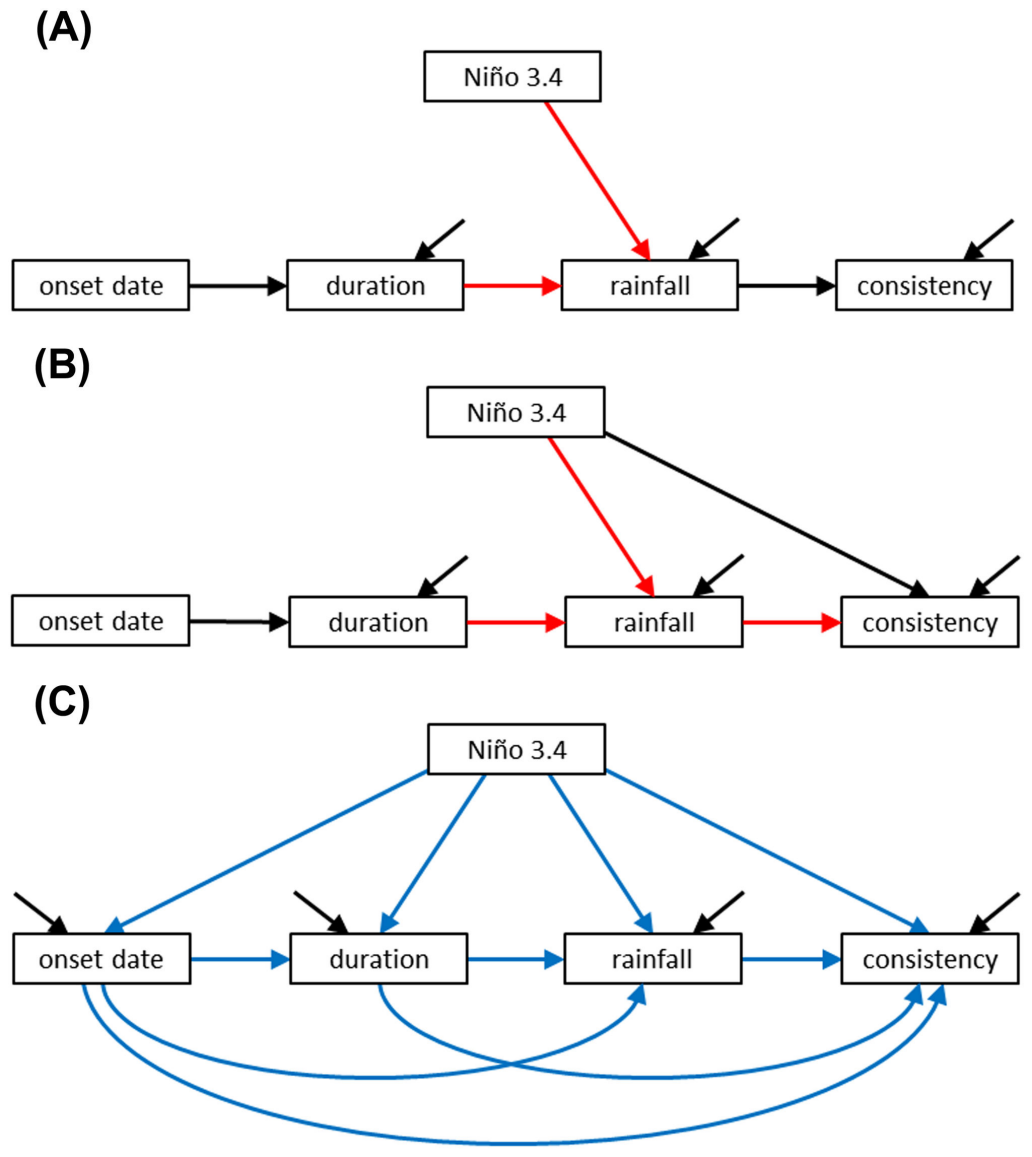
Conceptual causal diagrams describing relationships among descriptors of ENSO and the seasons. We designed these models for (A) the dry season and (B) the wet season based on relationships that have support from previous studies (see text). Arrows are coded to represent predicted effects (black = negative effect, red = positive effect). Using these starting models as a basic framework, we designed a third model (C) which specified all possible relationships among the seasonal descriptors (blue arrows). This latter model was saturated and was used to produce exploratory models using Amos’s model specification procedure. In all panels, diagonal black arrows represent error terms.

Our starting models for the dry and wet seasons are shown in Figure 1A and 1B, respectively. Our reasoning for the pathways of these models is:

1 *Seasons starting earlier in the year will be longer and have more rainfall*. These are straightforward relationships that are supported by correlations in the studies by Goswami and Xavier [6] and Camberlin and Diop [8].
2 *Heavy rainfall will reduce the consistency of the drying trend in the dry season and increase the consistency of the moistening trend in the wet season*. A graphical analysis in Slocum et al. [2] found that dry seasons with more rainfall had lower trend consistency. For the wet season, we reasoned that more rainfall would reduce the possibility of droughts.
3 *ENSO will influence rainfall, particularly in the dry season*. For dry seasons in Florida it has been well documented that rainfall increases under El Niño and decreases under La Niña [10,23-28]. These effects were confirmed for our specific site in Slocum et al. [2]. For the wet season, Slocum et al. [2] also found a weak positive relationship between Niño 3.4 and rainfall, so we specified such a relationship in Figure 1B. We note, however, that other studies have found that El Niño has a weak tendency to induce drought during the wet season [29,30].
4 *El Niño conditions will negatively affect trend consistency in both seasons*. In Florida, the increased dry season rainfall induced by El Niño occurs because El Niño allows greater passage of frontal systems through the region [26,28,31,32]. In Slocum et al. [2] these systems appeared as peaks of CRAs in the graphical analysis mentioned above. We therefore predicted that dry seasons undergoing El Niño conditions would have lower trend consistency, and that this effect would be routed via rainfall (i.e., we expected the effect to be indirect) (Figure 1A). Conversely, we expected that La Niña conditions would produce the opposite results. For the wet season, we also predicted that El Niño conditions would decrease trend consistency, as such an effect was found to be strong in the previous study. This prediction is also consistent with the results of other studies indicating that El Niño induces drought in the wet season [29,30]. We did not specify this relationship as routing via rainfall (as we specified the relationship between Niño 3.4 and rainfall to be positive and such a positive relationship cannot “conduct” a negative one). Rather, we specified a direct negative relationship between Niño 3.4 and wet-season trend consistency in Figure 1B.

Note that by putting our starting models together in these ways we purposely produced models that were both simple and theoretically consistent. Such an approach, however, constrains the models to contain indirect pathways that do not necessarily have theoretical support. For example, our dry season model (Figure 1A) suggests that longer dry seasons will have less consistent drying because they have more rainfall. Models that only contain such indirect pathways constitute what are known as “mediation tests”, that is, the models assume that all of the important variation between two variables is accounted for by their relationships with a mediating variable. If this assumption does not hold, then model evaluation will indicate poor goodness of fit. Such meditating effects are one reason to follow up starting/conceptual models with more exploratory efforts.

For our exploratory models, we used the same general framework as used in the starting models, but we specified all possible relationships within this framework so that the model became saturated (Figure 1C). We then ran a model selection process that examined all possible combinations using these pathways (described in more detail below). We predicted that this process would produce models that would confirm many of the pathways outlined in our starting models, but could also specify new pathways of interest. When new pathways were specified, we carefully considered if they were theoretically likely in the Discussion.

Finally, readers may note that the models in Figure 1 contain onset date and not cessation date. The reason for this is that cessation and onset date are used to calculate duration, and thus these two variables do not contain unique information when included together in a model with duration. We therefore (and quite arbitrarily) decided to include onset date and not cessation date in our models. It is still of interest, however, to understand how cessation date may influence the other model elements. We therefore conducted another set of analyses involving cessation date. The hypotheses tested were parallel to those used for onset date, that is: are seasons that end later in the year longer, and how does ENSO influence cessation date and duration, and via these two variables, rainfall? For the sake of brevity, we relegate these analyses to Appendix S2.

### Wildfire models

**Figure 2:**
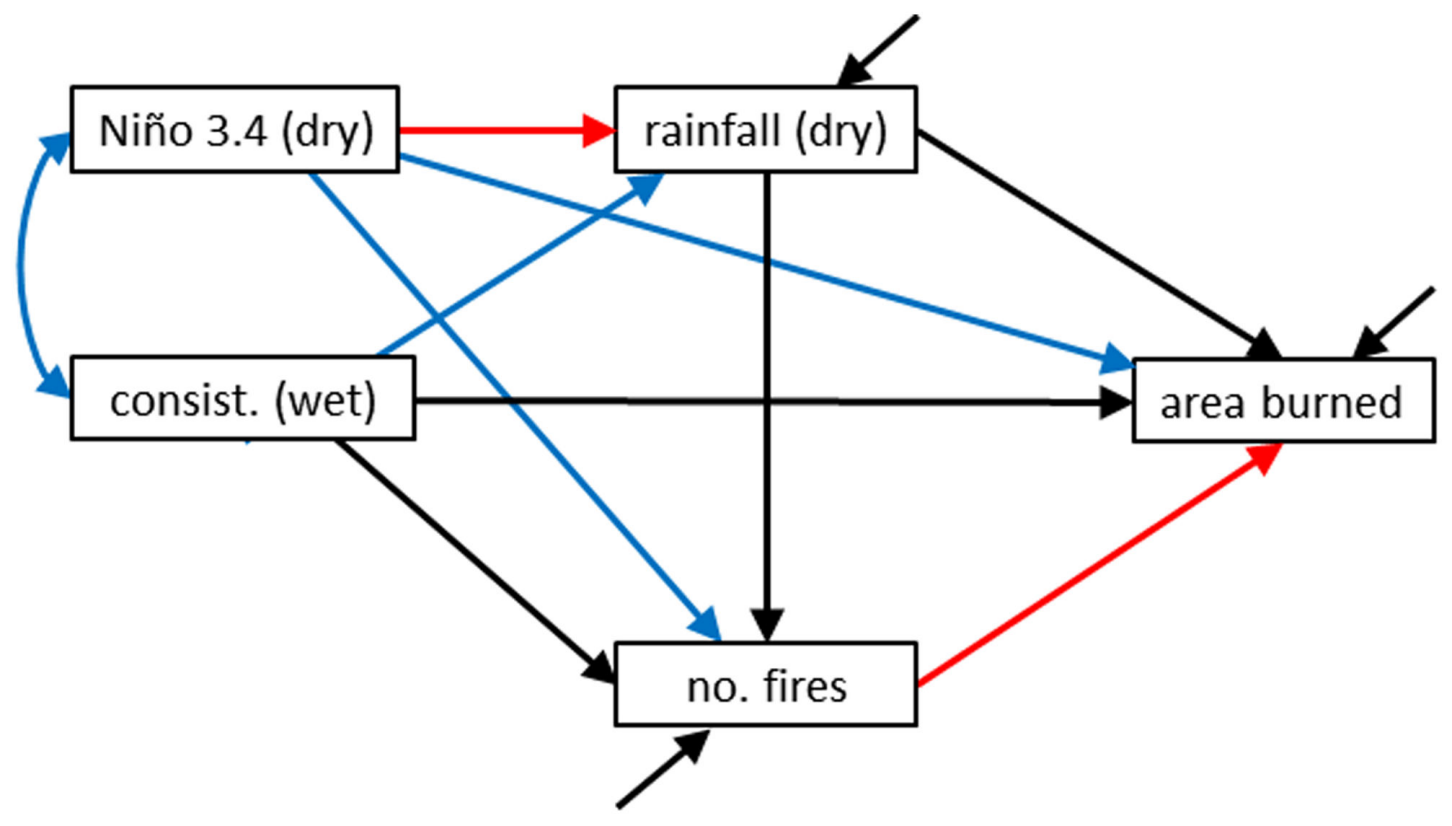
Conceptual model describing how ENSO and seasonal climate govern the wildfire regime. Included in the model are climate variables found to be important in the previous study [2], including Niño 3.4 (measured over the dry season), dry-season rainfall, and the consistency of the moistening trend in the previous wet season. Black and red arrows represent predicted negative and positive effects, respectively, and together make up our starting, theory-based model. Blue arrows were then added to the framework to saturate it for use in exploratory models produced by Amos’s model specification procedure.

Our starting model describes how wildfire at the APAFR may have been influenced by ENSO and seasonal descriptors (Figure 2). Because of our small sample size (n = 30 years), it was important to make this model as simple as possible. We therefore only included seasonal descriptors that were found to be the most important in Slocum et al. [2] for affecting wildfire area burned and number of fires. The specific pathways we included in the model were:

1 The positive effect of Niño 3.4 on dry season rainfall.
2 A negative effect of dry season rainfall on area burned and number of fires [10-12,33].
3 A positive effect of number of fires on area burned.
4 A negative effect of wet-season trend consistency on number of fires and area burned in the subsequent fire season. These relationships were found to be strong in the previous study using multiple regression [2].

Adding these pathways together in a model suggests that the following mediating relationships may be important (Figure 2)

5 By increasing rainfall, Niño 3.4 will reduce area burned and number of fires.
6 Wet seasons with consistent moistening will reduce area burned by reducing number of fires.
7 Dry season rainfall will reduce area burned by reducing number of fires.

For our exploratory models describing wildfire relationships, we used the same approach that we used for the seasonal models, that is, we took our starting model and added all the remaining pathways so that the model became saturated (Figure 2). We then tested all possible combinations of this saturated model using the model selection process.

### Data analysis

Before conducting SEMs, we examined the normality of all variables. In most cases the data were not normal, and we therefore applied transformations. To correct for positive skew, we applied either a square root transformation (wildfire number and area burned), a natural log transformation (wet season duration and rainfall), or a log base-10 transformation (dry season onset date). To correct for negative skew, we squared the data (onset date of the wet season and duration of the dry season). The frequency distribution for trend consistency had extreme negative skew, and we therefore “reflected” the data so that they became positively skewed, transformed them using the natural log transformation, and then re-reflected them [34]. This was accomplished by using this formula: *y*=(*abs*(ln(1-*x*))), where *y* is the transformed value, *abs* is absolute value, *ln* is the natural log function, and *x* = the *R* ^2^ score estimating trend consistency (see Appendix S1). The performance of these transformations was evaluated using box-and-whisker plots and Shapiro-Wilk tests using the UNIVARATE procedure of SAS release 9.3 (SAS Institute Inc., Cary, North Carolina). We also examined the relationships in our SEMs to see if they were linear. After the transformations, all relationships were found to be linear.

Estimation for our SEMs was conducted using maximum likelihood. Model fit was based on χ^2^ values and their associated *p* values. *P* values ≤ 0.05 indicate that the χ^2^ score rejects the null hypothesis stating that there is no difference between the underlying pattern in the data implied by the model and the underlying pattern found in the raw data (with “underlying pattern” referring to the respective correlation matrices). Thus, rejecting the null hypothesis indicates that a SEM fits the data poorly [22]. In the models, the strength of the various effects (arrows) are shown with path coefficients. We used partial regression coefficients, which represent the change expected if a predictor is varied and the rest of the predictors in the model are held constant. We standardized these coefficients so that they were readily comparable to each other (i.e., they are presented in standard deviation units). The collective ability of the coefficients to explain variation in the endogenous variables are shown with *R* ^2^ scores. All analyses were conducted using IBM SPSS Amos version 21 [35].

The exploratory models were created and evaluated using the specification search procedure of Amos. This procedure examined all possible combinations of the pathways shown in Figures 1C and 2, producing a list of models that can be sorted by various fit statistics. We found the Browne-Cudeck criterion (BCC) to be the most useful (see 30). This statistic is similar to the Akaike Information Criterion (AIC) in that it measures the trade-off between model complexity and model fit. “Good” models are simple but still have high goodness of fit, and are given a lower BCC. The BCC imparts a somewhat stronger penalty than the AIC if the model is complex [36]. We examined the model with the lowest BCC value (i.e., the “best” model), and then the next nine models with the lowest BCC values. For each of the seasonal models we found that the “best” model was the most informative, so we do not describe the other models. For the wildfire models we found that the second “best” model was of most interest, and we therefore detail it and contrast it with the “best” model.

We performed diagnostic procedures to address issues with autocorrelation and limited sample size [37]. These diagnostics revealed no substantial issues, and are detailed in Appendix S3.

## Results

### Seasonal models

**Figure 3:**
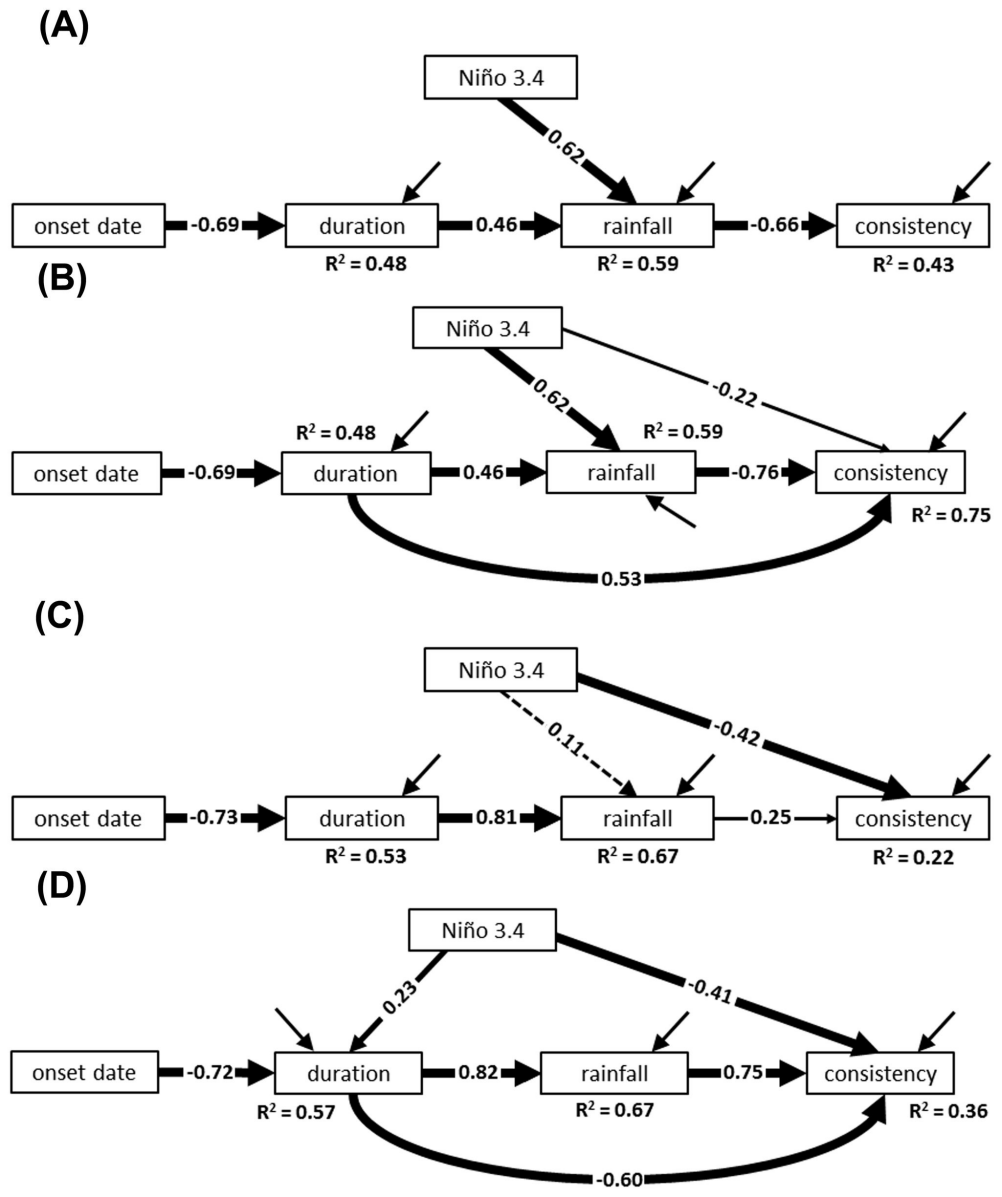
Structural equation models describing relationships among ENSO and seasonal characteristics for the two seasons. Models include (A) our starting, theory-driven model for the dry season (BCC = 65.6; χ^2^ = 45.3, df = 6, *p* < 0.001), (B) the “best” exploratory model for the dry season according to BCC values (BCC = 39.7; χ^2^ = 3.7, df = 4, *p* = 0.44), (C) our starting model for the wet season (BCC = 40.6; χ^2^ = 18.2, df = 5, *p* = 0.003), and (D) the “best” model for the wet season (BCC = 28.8; χ^2^ = 4.1, df = 4, *p* = 0.40). Paths are accompanied by numbers, which are standardized partial regression coefficients. The significance of these coefficients is shown with differently weighted lines (dashed = non-significant, thin = *p* ≤ 0.05, medium = *p* ≤ 0.01, and thick = *p* ≤ 0.001). Models use 58 years of climate data collected for the Avon, Park Air Force Range, south-central Florida, USA (1950-2007).

We began by examining the dry season as outlined in our starting model (Figure 1A). Maximum likelihood estimation of this model produced a BCC of 65.5 and a χ^2^ of 45.3 with 6 df (*p* < 0.001) (Figure 3A). This model was therefore indicated to fit the data poorly, suggesting that our model based purely on theoretical evidence was not sufficient to describe all the important patterns in the underlying data.

We therefore ran our exploratory procedure (Amos’s specification search) in which we allowed the data to specify “competing” models within the general framework of the starting model (Figure 1C). The “best” model produced by this procedure (i.e., the model with the lowest BCC value) had a BCC of 39.7 and a χ^2^ of 3.7 with 4 df (*p* = 0.44) (Figure 3B). This model was therefore indicated to fit the data and be safe to interpret. It contained all of the pathways of the starting model, and indicated that they were all very strong (*p* values ≤ 0.001). Rainfall had the expected negative effect on trend consistency (i.e., dry seasons with more rainfall were “punctuated” with more storms), and onset date had the expected negative effect on season duration (i.e., dry seasons were shorter when they started later in the year). Onset date also had an indirect negative effect on rainfall. The strength of this effect can be calculated simply by multiplying the relevant pathways (that is, -0.69 × 0.46 = -0.32). Thus, the model suggested that dry seasons starting later in the year had less rainfall because they were shorter. Finally, Niño 3.4 induced rainfall, and, as expected, this produced an indirect negative effect on trend consistency (0.62 × -0.76 = -0.47).

The model, however, outlined two trends that were not specified in the starting model (Figure 3B). The first was a direct negative effect of Niño 3.4 on trend consistency; this effect was statistically significant but fairly weak. The second was a direct effect of duration on trend consistency (path coefficient = 0.53). This effect canceled out much of the indirect negative effect of duration on trend consistency that routed via rainfall (0.46x -0.76 = -0.35), such that the total effect was 0.18 (= 0.53 + -0.35). The model therefore indicated that longer dry seasons had a slight tendency to have a more consistent drying trend.

We examined the effect of dry-season cessation date in a separate analysis in Appendix S2. In this analysis, we examined a model that was similar to the “best” model described above, but which used cessation date instead of onset date. The expected relationships were revealed: dry seasons that ended later in the year were longer (path coefficient = 0.53, *p* ≤ 0.001, *R* ^2^ = 0.28) and had more rainfall (indirect effect via duration: 0.53 × 0.46 = 0.23).

We next turned to the models describing the wet season. Our starting model, as outlined in Figure 1B, had a BCC of 40.6 and a χ^2^ of 18.2 with 5 df (*p* = 0.003) (Figure 3C). Therefore, this model, like the starting model for the dry season, was also indicated to fit the data poorly. We therefore continued our analysis by producing exploratory models (Figure 1C). The resultant “best” model is shown in Figure 3D (BCC = 28.8, χ^2^ = 4.1, df = 4, *p* = 0.40). Compared to the starting model, this new model indicated no effect of Niño 3.4 on rainfall, but Niño 3.4 had a positive effect on duration. Like in the dry-season model, duration was specified to have a direct relationship with trend consistency, but in this case it was negative. All of these effects were statistically significant. In addition, the coefficient for the pathway from rainfall to trend consistency changed from 0.25 in the starting model to 0.75 in the “best” model. Otherwise, the two models were similar, having nearly identical coefficients for the pathways between onset date and duration, duration and rainfall, and Niño 3.4 and trend consistency.

We interpreted the “best” wet-season model by starting with the triangle of relationships among season duration, rainfall, and trend consistency. This triangle specified the expected positive effect of rainfall on trend consistency (path coefficient = 0.75), that is, rainier wet seasons had more consistent moistening. Also, season duration was found to have a positive indirect relationship with trend consistency by increasing rainfall (0.82 × 0.75 = 0.62), but this effect was almost entirely cancelled out by the direct negative effect (path coefficient = -0.60). Thus, the model indicated that longer wet seasons were not any more consistent in their moistening trend than shorter wet seasons.

Onset date was shown to have a strong negative effect on duration, and via this route it also had a strong indirect effect on rainfall (-0.72 × 0.82 = -0.59). Wet seasons that started later in the year tended to be shorter and have less rain.

In examining the effects of cessation date (Appendix S2), we found that, as with the dry season, wet seasons that ended later in the year were longer (path coefficient = 0.76, *p* ≤ 0.001, *R* ^2^ = 0.58) and had more rainfall (indirect effect via duration: 0.76 × 0.82 = 0.62). We also found, however, that the most relevant model was one that included a pathway from Niño 3.4 to cessation date (path coefficient = 0.35, *p* = 0.006, *R* ^2^ = 0.12) and not one from Niño 3.4 to duration. This result suggests that the way Niño 3.4 increased wet-season duration (as shown for the “best” model in Figure 3D) was by delaying when the season ended.

### Wildfire models

**Figure 4:**
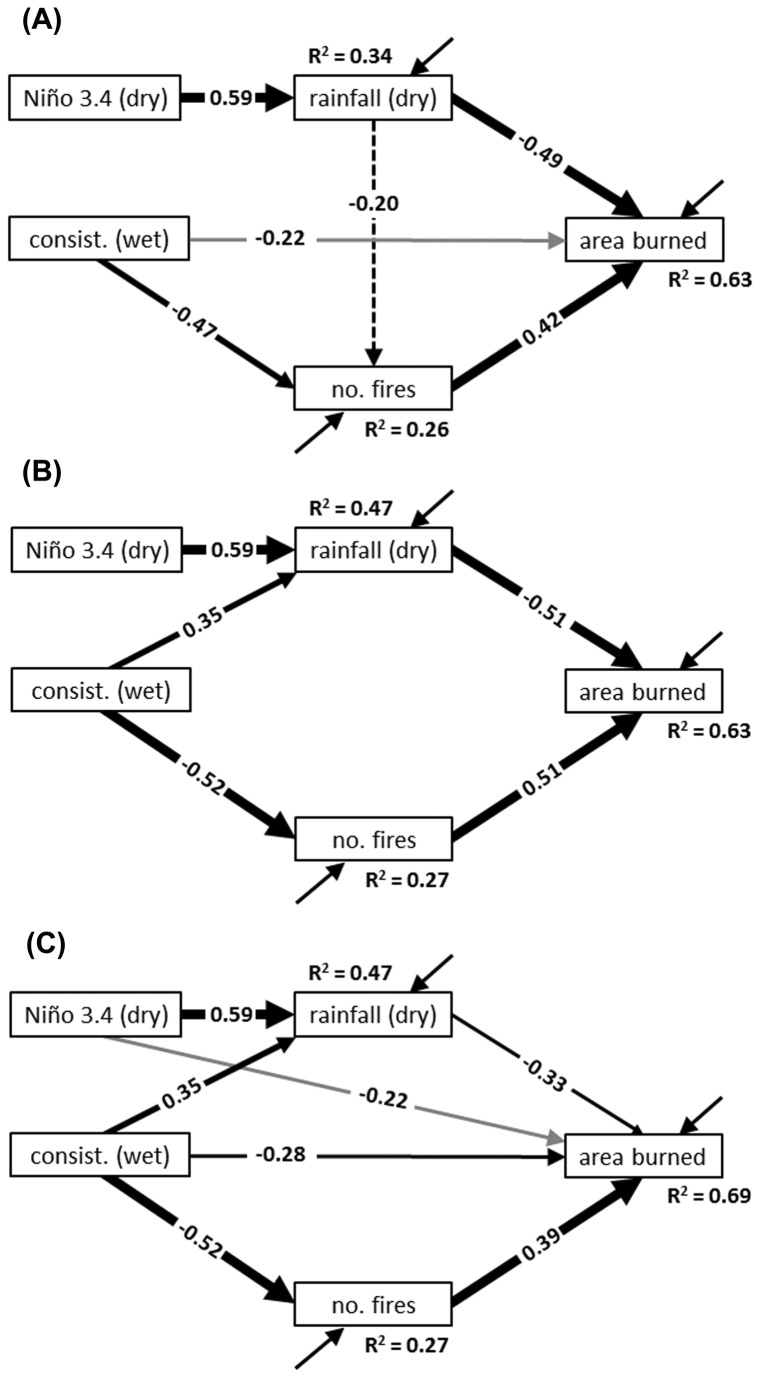
Structural equation models describing effects of selected climate variables on the wildfire regime. The models include: (A) our starting, theory-based model (BCC = 37.0; χ^2^ = 9.3, df = 4, *p* = 0.05), (B) the second “best” exploratory model according to BCC values (BCC = 32.4; χ^2^ = 7.1, df = 5, *p* = 0.21), and (C) the “best” exploratory model (BCC = 32.3; χ^2^ = 2.0, df = 3, *p* = 0.57). Paths are labeled with numbers, which are standardized partial regression coefficients. The significance of these coefficients is shown with differently weighted lines (dashed = non-significant, gray = *p* ≤ 0.10, thin = *p* ≤ 0.05, moderate = *p* ≤ 0.01, and thick = *p* ≤ 0.001). Models are drawn using 30 years of climate and wildfire data from the Avon, Park Air Force Range, south-central Florida, USA (1978-2007).

Our starting model had poor fit (BCC = 37.0; χ^2^ = 9.3, df = 3, *p* = 0.05; Figure 4A), and we therefore proceeded to explore other models using Amos’s specification procedure. We found that, of the top ten models produced by the procedure (as ranked by BCC values), the second “best” model was the most interesting because of its combination of simplicity and explanatory power (BCC = 32.4; χ^2^ = 7.1, df = 5, *p* = 0.21; Figure 4B). This model differed from the starting model by not including pathways from dry-season rainfall to number of fires and from wet-season trend consistency to area burned. Moreover, it included a strong positive pathway from wet-season trend consistency to dry-season rainfall. The inclusion of this pathway was the main reason the second model fit the data in contrast to the starting model.

Overall this model explained area burned by wildfires with high accuracy (*R* ^2^ = 0.63). Two strong direct effects contributed to this score: a negative effect of rainfall and a positive effect of number of fires. Area burned was also reduced by three indirect effects: it was reduced when Niño 3.4 induced more rainfall (0.59 × -0.51 = -0.30), when consistent moistening in the previous wet season reduced number of fires (-0.52 × 0.51 = -0.26), and when consistent moistening in the wet season was followed by rainier dry-seasons (0.35 × -0.51 = -0.18).

The model explained number of fires with less accuracy (*R* ^2^ = 0.27). Number of fires was shown to be reduced when the previous wet season had greater trend consistency. Surprisingly, number of fires was not significantly affected by dry-season rainfall (a result also found in the starting model; Figure 4A). This meant that the model failed to support our prediction that dry-season rainfall would reduce area burned by reducing number of fires.

The “best” model was more complex than the second “best” model (BCC = 32.3; χ^2^ = 2.0, df = 3, *p* = 0.57; Figure 4C). It indicated two additional negative effects on area burned, one from Niño 3.4 and the other from wet-season trend consistency. These additional pathways were accompanied by a weaker pathway from dry-season rainfall to area burned. This configuration boosted the model’s *R* ^2^ score for area burned in comparison to the other two models.

## Discussion

### Patterns in seasonal rainfall

In our SEMs exploring seasonality, we used a model building approach in which we started with models whose pathways had theoretical evidence, and followed with models that were more exploratory. Here we follow this approach with a discussion of which pathways in the models have the strongest evidence for causality, and which have the weakest (and should therefore be considered hypothesis generating). Again, we caution that, because our data are observational, by “causal” we mean relationships that have theoretical or logical evidence to be so.

The mechanisms with perhaps the strongest causal evidence were those involving how ENSO affected the dry season. Dry seasons with positive Niño 3.4 values have more rainfall, a finding fully consistent with the literature [2,10,23-28]. This additional rainfall interrupts the season’s drying trend. El Niño events result in low pressure over the southeastern United States during winter/early spring, allowing the jet stream to move farther south and to bring with it more storm fronts [26,28,31,32]. Conversely, La Niña events induce higher pressure, steering the jet stream away from the region and allowing consistent desiccation of the region’s fuels during the dry season.

Another set of relationships that we view as having strong support for causality were those involving onset date, cessation date, duration and rainfall: logically, seasons that started later in the year (or end earlier) were shorter and had less rainfall. Similar results were found in the correlation analyses conducted by Camberlin and Diop [8] for Senegal and by Goswami and Xavier [6] for India.

In examining the effect of season duration on trend consistency, our models indicated no net effect for the wet season and a weak positive effect for the dry season. We interpreted this to mean that longer seasons were just as likely to be interrupted by an event (e.g., a storm front in the dry season or a drought in the wet season) as they were to have some continuous pattern that reinforced the trend. We note that our starting models did not specify direct pathways between duration and trend consistency; rather, they proposed that duration’s effect on trend consistency would route via rainfall. This proposed indirect mechanism, however, was not supported by the data, and was the main reason that the starting SEMs had poor fit.

One set of hypothesis-generating relationships revealed in the analyses involved how ENSO affected the consistency of the drying and moistening trends. Trend consistency is a unique variable derived in the previous study [2], and as such relationships involving it should be regarded as hypothesis generating. For the dry season – while we found that Niño 3.4 negatively affected trend consistency by producing more rainfall – there was also some residual negative effect that required a direct pathway between Niño 3.4 and dry-season trend consistency. What generates this effect is unknown, but may have something to do with how ENSO affects the transitions among the seasons (e.g., are they gradual or more abrupt under particular ENSO phases?). For the wet season, our models revealed a strong negative effect of Niño 3.4 on trend consistency, suggesting that El Niño conditions were inducing droughts. This agrees with other studies that found that El Niño can induce drought in the wet season [29,30] by producing greater wind shear at upper levels of the atmosphere, thereby reducing the formation of tropical storms [38,39]. We note, however, that our models did not find a negative relationship between wet-season rainfall and Niño 3.4. Thus, our analysis suggests that ENSO had a stronger effect on rainfall variation in the wet season than it had on rainfall amount at our site.

El Niño conditions were indicated to delay cessation of the wet season, thereby lengthening the season and leading to more rainfall. Other studies have also found that ENSO affects wet season duration and rainfall. Lima and Lall [7], working in northeastern Brazil, found that El Niño delayed onset of the wet season, leading to drought. Similar results were found for India by Goswami and Xavier [6], where El Niño “squeezed” the wet season by delaying onset and hastening cessation. We view our finding – while being similar to those of other studies – to be primarily hypothesis generating, as more study is required to determine the mechanism via which this delay of cessation takes place.

Overall, our study shows how SEMs can be used to take full advantage of the rich data sets provided by the sophisticated techniques recently developed to define seasons (e.g., [6-8]). In particular, SEM allows mediation tests. For instance, our SEMs allowed us to determine if the effect of ENSO on rainfall appeared to be mostly direct, or to be stronger when routed via duration. The determination of such a mediating mechanism, however, cannot be determined when using univariate analyses. In the correlations provided by Goswami and Xavier [6], for example, it appears that the way ENSO affected rainfall was via its effect on duration, but the appropriate partial regression coefficients were not examined, and so the real strength of this mechanism remains unclear. More importantly, such univariate approaches leave students of ENSO and seasonality left to their own devices to derive what the causal mechanisms might be; no specific causal structure is proposed. This lack of specificity in the proposed theory makes it difficult to agree with it or oppose it, and thereby develop new theory.

### Patterns in the wildfire regime

The strongest and simplest set of pathways describing how seasonal climate and ENSO affected the APAFR’s wildfire regime were found in the SEM shown in Figure 4C (the second “best” model as suggested by BCC values). We view these pathways as either having strong evidence for causality, or as detailing important hypothesis-generating relationships. The clearest causal pathway we found described how ENSO, by affecting rainfall, influenced area burned. The previous work conducted at the site [2] showed that ENSO influenced rainfall, and, in a separate univariate analysis, that rainfall influenced area burned. This suggested a mediation model. SEM clearly shows that this mediation model is correct, and estimates the strength of the relationships. This mechanism of ENSO affecting wildfires by governing rainfall has been well documented in southern Florida [2,10,40,41], and parallels a worldwide trend [42]. However, like Slocum et al. [2], these previous studies fail to detail the mechanism using a causal network, but instead rely on univariate analyses.

Another straightforward causal relationship incorporated in the model detailed the effect of fire frequency on area burned. This relationship was shown to be statistically significant, but was not as strong as one might suspect (it accounted for 25% of the variation in area burned; see Figure 4B). This finding reflects how area burned at the APAFR is not governed so much by number of wildfires as it is by large wildfires. For example, just five wildfires accounted for one third of the total area burned over 1997-2007 [43]. The greater importance of large fires versus number of fires for explaining total area burned is a general characteristic of wildfire regimes [44-46].

One hypothesis-generating relationship detailed in the SEM described how consistency of moistening in the wet season reduced number of fires and area burned in the next fire season. We suspect that wet seasons with consistent moistening ended with a larger buildup of moisture in the system (in fuels, soils, marshes, and so forth) compared to wet seasons that had droughts. This buildup meant that more time was required for fuels to become sufficiently dry to be ignited in the subsequent wildfire season. In particular, such seasons may have prevented military missions from igniting fires in January, February and March. These results suggest that our study should be followed up with studies of how wet-season trend consistency relates to fuel moisture, run off, and water levels in different habitats, especially those habitats that break up fuel continuity in the region (e.g., marshes) (see 47). In addition to this effect on number of fires, wet seasons with consistent rainfall tended to be followed by dry seasons that were rainier. Again, this suggests a set of follow-up studies, for example, an examination of how trend consistency affects the transitions between the seasons.

Finally, the model revealed one pattern that was found to be non-significant statistically but which theoretically should be causal: the effect of dry season rainfall on number of fires. Certainly this is a relationship that is a general characteristic in most fire-prone systems [44], and a statistically significant relationship was found in a study conducted for the nearby Everglades National Park [10]. We therefore postulate that this relationship would be revealed to be statistically significant if more years of data were collected.

The “best” wildfire model suggested by BCC values, besides fitting the underlying data better than the other tested models, also produced a higher *R* ^2^ score for area burned. However, we found this model to be overly complex by its inclusion of pathways from Niño 3.4 and wet-season trend consistency to area burned. Besides being of low statistical significance, these pathways had less evidence for causal support than the pathways found in the second “best” model. Therefore, we viewed these pathways as hypothesis generating, that is, they suggest that there may be additional mechanisms by which ENSO and the previous wet season influence wildfire activity besides through their effects on number of fires and dry-season rainfall.

In summary, like with our seasonal models, our SEM analysis of our climate/wildfire data produced a more complete picture of the system than was presented in the univariate analyses conducted in the previous study [2]. In particular, it outlines mediation effects that cannot be described using multiple regression. We note, however, that because our fire model has only 30 years of data, it was only capable of outlining the strongest trends, and much of the work it performs is hypothesis generation. Also, because of limited sample size we did not analyze many variables that may have generated interesting effects (e.g., dry season duration, which was indicated to be important in the previous study [2]). We made this decision to reduce the number of variables to avoid the serious error of over-fitting our model. With more years of data, however, it will be possible to examine the effects of more variables, and thereby tie together the fire model with the seasonal models more closely.

## Conclusions

When seasons are fully characterized using sophisticated methodologies, rich data sets are produced that hold much promise for determining how seasonality affects ecological processes. However, the analysis of such data sets requires statistical techniques that properly estimate the relationships – particularly the causal ones – among the various seasonal descriptors derived. We found that when we took such a data set of seasonal descriptors, and analyzed it with SEM, we were able to effectively describe how the seasons were influenced by ENSO, and how in turn seasonal climate regulated a wildfire regime. While our models were limited in several important ways – they had limited sample size, and could draw from only a handful of other studies to derive theory – the causal networks proposed are, in our opinion, much more efficient for matching data to theory than the univariate analyses performed in the previous study [2].

We view our results as being informative for two particular sets of ecological studies. The first set are studies that use SEMs to describe how climate affects ecological systems. Perhaps the field where this is most developed is population ecology (e.g., [48-51]), but similar models are also starting to be developed in community ecology [52-54] and disturbance ecology [55]. We propose that these studies could benefit by incorporating more sophisticated descriptors of seasonality into their SEMs. The second set of ecological studies are those that use a more thorough parameterization of seasonality. These studies include – as mentioned in the Introduction – those that use animal behavior to estimate biologically relevant seasonal descriptors [1,5], as well as studies on wildfires [2,3]. These studies could benefit from using SEM or other causal modeling. In short, we advocate that studies investigating how seasonality affects ecosystems could benefit from a “combined arms” approach that uses both SEM and more advanced techniques for defining seasons. Our study demonstrates that this approach works well in not only describing the potential causality among the various descriptors of seasonality and wildfire, but also for outlining hypotheses for future study. For example, the approach may prove effective in predicting how climate change might affect the seasons, perhaps by affecting global climate cycles [56], which in turn may cascade to affect ecological processes such as disturbance (cf. [3]).

## Supporting Information

Appendix S1 **Calculation and use of cumulative rainfall anomalies.**
(DOC)

Appendix S2 **Modeling of cessation date.**
(DOC)

Appendix S3 **Diagnostic procedures for structural equation modeling.**
(DOC)
