# Supplementary material for: A Structural Equation Model Analysis of Relationships among ENSO, Seasonal Descriptors and Wildfires
Source: PLoS One. 2013 Sep 24;8(9):e75946. doi: 10.1371/journal.pone.0075946 (PMC3782436; doi:10.1371/journal.pone.0075946)
Supplement: Appendix S1 — Calculation and use of cumulative rainfall anomalies. (DOC) [file pone.0075946.s001.doc]

**Appendix S1**

The calculation of cumulative rainfall anomalies (CRAs) is relatively straightforward. Given a time series of daily rainfall data, the following steps are taken: (1) the mean of the entire time series is derived, (2) this mean is subtracted from each day of record (producing daily rainfall anomalies), and (3) the anomalies are added together consecutively starting from the first day of record (producing cumulative rainfall anomalies). There are some additional details in these calculations. It is generally useful, for example, to transform the data to reduce the influence of extreme rainfall events. Also, if one wishes to describe a general pattern of rainfall over a region, it is important to use more than one rainfall station, as one station can be heavily influenced by spatial variation. See Slocum *et al.* [2] for additional details, and Camberlin and Diop [8] for an example that uses principal components analysis.

This transformation of daily rainfall data is useful because it converts the data into a waveform, allowing the ready visualization of temporal patterns. Dry seasons are characterized by consistent decreases in CRAs, the result of consecutively adding negative rainfall anomalies, and wet seasons are characterized by consistent increases in CRAs, the result of adding positive anomalies. Where these downward and upward trends meet within a year is used to define the date of onset of the wet season (the cessation date of the dry season). Similarly, the onset date of the dry season (cessation date of the wet season) is estimated where the upward trend of the wet season meets the downward trend of the dry season.

In Slocum *et al.* [2] CRAs were used to define the wet and dry seasons from 1950 to 2007 at the Avon Park Air Force Range. An example is shown below (Figure S1-1) for the years of 1951 and 1952. The upward arrow indicates the onset date of the wet season of 1952 (and cessation date of the dry season), and the downward arrows indicate cessation dates of the wet seasons of 1951 and 1952 (and onset dates of the dry seasons).


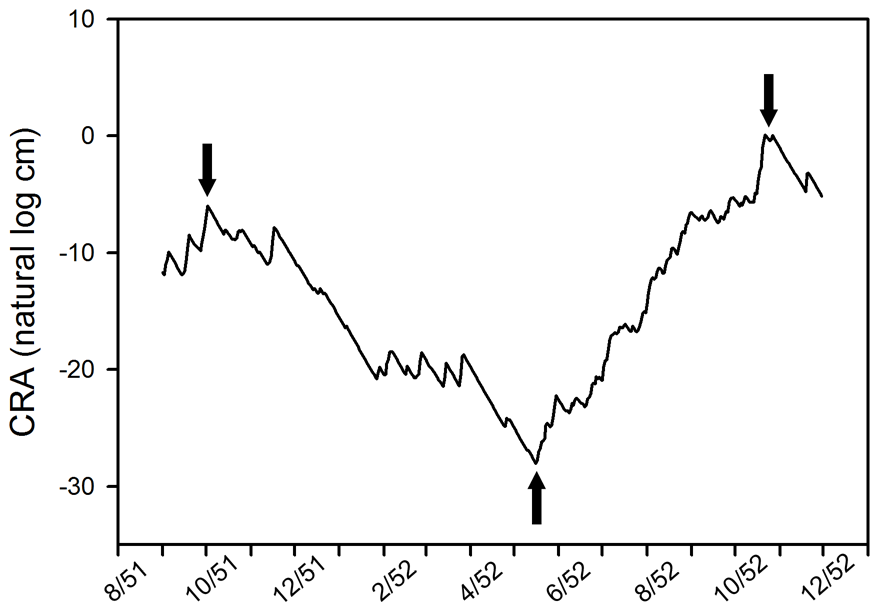


**Figure S1-1.** **Example of how cumulative rainfall anomalies (solid black line) help define wet and dry seasons.**

Once onset dates are defined, it is easy to derive other characteristics describing the seasons. Duration of a season is simply calculated from onset and cessation dates. Total rainfall can also be estimated; such an estimate is more accurate than using a conventional definition of season based on months (e.g., June 1^st^ to September 30^th^) (see Slocum *et al.* [2] for details of the consequences of this lack of accuracy). A third more complicated seasonal characteristic is “trend consistency”. This is a measure of how consistent the drying trend is during the dry season and the moistening trend is during the wet season. Trend consistency for a particular season is measured using the *R^2^* score derived from a linear regression that uses day of the year as the independent variable and CRA as the dependent variable (e.g., the dashed red lines in Figure S1-1). Trend consistency turned out to be an important variable for describing relationships between wildfire activity and seasonal rainfall at the study site [2].
