# Supplementary material for: A Structural Equation Model Analysis of Relationships among ENSO, Seasonal Descriptors and Wildfires
Source: PLoS One. 2013 Sep 24;8(9):e75946. doi: 10.1371/journal.pone.0075946 (PMC3782436; doi:10.1371/journal.pone.0075946)
Supplement: Appendix S2 — Modeling of cessation date. (DOC) [file pone.0075946.s002.doc]

**Appendix S2**

Like with any general linear model, SEMs that include variables that are linear combinations of each other will not perform correctly. Thus duration, onset date, and cessation date cannot be included together in a model, as duration is calculated from (is a linear combination of) onset and cessation dates. Because of this situation, in the manuscript we elected to keep onset and duration in the models, and to detail the results with cessation date in this appendix. Note, however, that we could just as easily have done the reverse and presented the results with cessation in the manuscript and onset in the appendix. Our analyses of the data suggests that both approaches produce similar patterns and interpretations.

The first point we make here is that a separate analysis of cessation date is only necessary because onset date, cessation date[[1]](#footnote-2), and duration are transformed to achieve normality. This is because if the data were not transformed, then onset and cessation dates will predict duration with perfect accuracy (i.e., with an *R^2^* of 1.0), and therefore any variation in duration not explained by one of the date variables must be explained by the second date variable. One could thus could very simply calculate the effect of the second date variable using this remaining variation. Take, for instance, our SEM for the dry season shown in Figure 3B. If this model had used non-transformed data instead of transformed data, then the *R^2^* score describing the amount of variation in duration explained by onset date (0.48) indicates that cessation date must explain the remaining variation (i.e., with an *R^2^* score of 0.52). This would give cessation date a path coefficient of $\sqrt{0.52}$, or 0.72. But the data are, of course, transformed, and no longer predict duration with perfect accuracy, and so this calculation cannot be used. It is therefore necessary to examine the effects of the two date variables in separate model. Finally, we note that despite the transformations “messing up” the linear combination, it is still not appropriate to include the two date variables in the same model, because from a theoretical standpoint they still do not contain unique information.

What then are the effects of cessation date on the dry and wet seasonal “systems”? First, we go over two hypotheses. The first hypothesis covers the obvious prediction that seasons will be longer if they end later in the year (as shown in Goswami and Xavier [6] and Camberlin and Diop [8]). Our second hypothesis is that ENSO may affect cessation, and in this way affect duration and rainfall [6,8]. One corollary with this second hypothesis is that, because duration consists of the information from onset and cessation dates, any effect of ENSO on duration has to consist of an effect on one or both of the date variables. This means that if a model indicates an effect of ENSO on duration, and not on the date variable included in the model, then the model is actually indicating an effect of ENSO on the date variable *not included*. The SEM in Figure 3D is therefore suggesting that ENSO is affecting cessation date of the wet season. Below we show if this line of reasoning is verified.

To test the effects of cessation date, we used the same procedures that we used to test the models in the manuscript, except we replaced onset date with cessation date in all of the SEMs. Below we present just a few of the results of this analysis. For the dry season, we present the second “best” model according to BCC values (Figure S2-1A). This model had a BCC of 27.2 and a χ^2^ of 2.5 with 4 df (*p* = 0.65). Its pathways are very similar in strength to those found for

**(B)**

**(A)**

**Figure S2-1.** **Structural equation models describing seasonal relationships when cessation date is included instead of onset date.** Shown are results for (A) the dry season and (B) the wet season. The significance of the path coefficients is shown with differently weighted lines (thin = *p* ≤ 0.05, medium = *p* ≤ 0.01, and thick = *p* ≤ 0.001).

the model using onset date (Figure 3B). The model clearly supports the hypothesis that dry seasons that end later in the year are longer and have more rainfall. (Note that the “best” model is essentially the same as the one presented, but it specifies a non-significant pathway from ENSO to cessation date.)

For the wet season, we found that the “best” model (Figure S2-1B) was more or less equivalent to the model using onset date in the manuscript (Figure 3D). This model had a BCC value of 28.6 and a χ^2^ of 3.9 with 4 df (*p* = 0.42). It clearly indicates that wet seasons that ended later in the year were longer and had more rainfall, and that El Niño conditions tended to reinforce this effect. This model also clearly supports our hypothesis that the mechanism via which Niño 3.4 lengthened the season was by delaying cessation date.

1. Cessation date of the dry season is transformed using a square transformation (to correct for negative skew), while the cessation date of the wet season is transformed using a log-10 transformation (to correct for positive skew). [↑](#footnote-ref-2)
