# Supplementary material for: A Structural Equation Model Analysis of Relationships among ENSO, Seasonal Descriptors and Wildfires
Source: PLoS One. 2013 Sep 24;8(9):e75946. doi: 10.1371/journal.pone.0075946 (PMC3782436; doi:10.1371/journal.pone.0075946)
Supplement: Appendix S3 — Diagnostic procedures for structural equation modeling. (DOC) [file pone.0075946.s003.doc]

**Appendix S3**

Regression type procedures such as SEM have a number of assumptions that, if violated, can result in biased regression coefficients and misleading interpretations. Our particular models have two potential issues, autocorrelation and limited sample size. Here we show how we successfully addressed these issues.

Autocorrelation

If there is a lack of independence among temporal sampling units (in our case years), it can result in a sample size that is effectively less than the starting sample size. For example, take a data set of 50 years of wildfire data in which area burned in one year is followed by less burning in the next year. In such a case the effective sample size may be less than the actual 50 years of data, as the data are not independent.

Amos lacks diagnostics to test for autocorrelation. This situation, however, is not difficult to remedy. All that is required is to set up multiple regression models that are equivalent to the particular parts of an SEM that one wishes to test. Autocorrelation is then dealt with by analyzing the residuals from these regressions. These methods are readily accomplished using procedures in software packages like SAS or R. Here we do them using the REG and AUTOREG procedures of SAS version 9.3 (SAS Institute Inc., Cary, North Carolina).

We first show how a multiple regression can be set up that describes a portion of a SEM. For our example, we use area burned as shown in our second “best” model describing the wildfire regime at the APAFR (Figure 4B). Below is a duplicate figure for the model (Figure S3-1), except that it shows non-standardized path coefficients and y-intercepts instead of standardized coefficients and *R^2^* scores:

**Figure S3-1. Structural equation model equivalent to that of Figure 4B, except with non-standardized path coefficients and y-intercepts displayed.**

From this SEM, the equation for calculating predicted values for area burned is derived using the direct arrows pointing to that variable, that is: $A=32.19-0.66r+7.37n,$ where *A* is area burned (square root transformed), *r* is rainfall (natural log transformed), and *n* is number of fires (square root transformed).

This equation can be reproduced in SAS using the REG procedure:

**proc** **reg** data=fire; model area = dryrain no; **run**;

In this statement, area represents area burned, dryrain is dry season rainfall, and no is number of fires. This procedure produced this output:


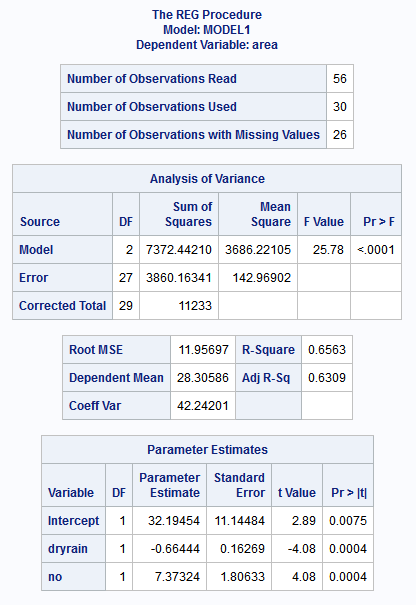


Here we see that the parameter estimates (black boxes) are the same as shown in Figure S3-1.

Given this, we proceeded to run an identical procedure, except one that tests to see if autocorrelation is present. The code below uses the SAS AUTOREG procedure to test for lagged effects up to four years (note that the data is sorted by year before starting the procedure):

**proc** **autoreg** data=fire;

model area = dryrain no / nlag=**4** method=ml dwprob;

**run**;

The output is:


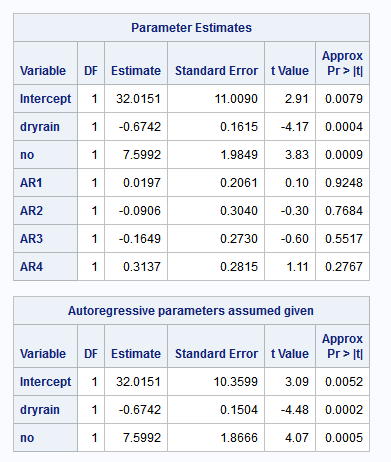


The lagged effects (AR1 to AR4) are all shown to not be statistically significant (gray boxes). The effects of dry season rainfall and number of fires, when adjusted for these non-significant lagged effects (black boxes), are only slightly different than the model that does not correct for autocorrelation.

A stepwise procedure for testing for lagged effects can also be conducted using the backstep option in the AUTOREG procedure:

**proc** **autoreg** data=fire;

model area = dryrain no / nlag=**4** backstep method=ml dwprob;

**run**;

This option deletes the lagged effects one-by-one if they are deemed non-significant. The output for this procedure is:


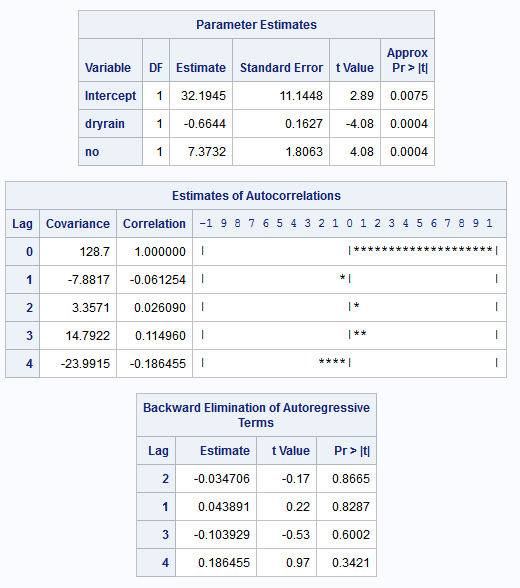


The output shows that the lagged effects were systematically eliminated, such that the parameter estimates for dry season rainfall and number of fires ended up being identical to those provided by our initial multiple regression and SEM models (Figure S3-1).

This set of examinations thus makes it clear that autocorrelation did not appear to be an issue for area burned. Similar results were found for all of the other endogenous variables in our SEMs.

Limited sample size

Our data set had low sample sizes relative to the complexity of the models being tested. For each model, the ratio (*d*) of sample size (*n*) to the number of unknown parameters being tested (*a*) was < 5. Based on this ratio, Lee and Song [37] recommend using Bayesian estimation to confirm the parameter estimates obtained using maximum likelihood.

Amos has a module that performs Bayesian estimation (see Chapter 26 in [35]). Here we show an example for the same SEM presented above for our analysis of autocorrelation (Figure S3-1). Running the Bayesian estimation for the model produces the following output:


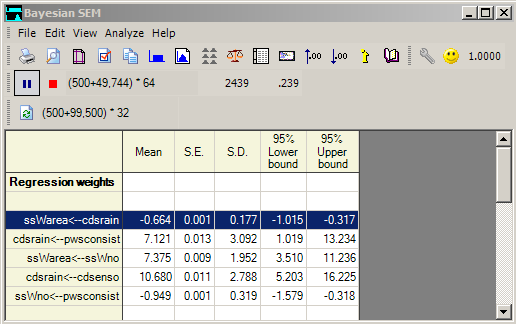


The procedure is run through as many iterations as needed for the model to reach convergence (shown with the smiling face). At this point the analysis can be stopped and the results interpreted. The various regression coefficients (non-standardized) are shown in the table. These estimates are nearly identical to those produced by the maximum likelihood model (Figure S3-1). For example, the coefficient for the pathway between area burned (ssWarea) and dry-season rainfall (cdsrain) is -0.664 for both the Bayesian and maximum likelihood estimations. The effect of previous wet-season trend consistency (pwsconsist) on number of wildfires (ssWno) is -0.949 using Bayesian estimation and -0.950 using ML.

Similar results were found for the other final models reported in the manuscript.
